# Responses to the Selective Bruton’s Tyrosine Kinase (BTK) Inhibitor Tirabrutinib (ONO/GS-4059) in Diffuse Large B-cell Lymphoma Cell Lines

**DOI:** 10.3390/cancers10040127

**Published:** 2018-04-23

**Authors:** Ryohei Kozaki, Meike Vogler, Harriet S. Walter, Sandrine Jayne, David Dinsdale, Reiner Siebert, Martin J.S. Dyer, Toshio Yoshizawa

**Affiliations:** 1 Department of Molecular and Cell Biology, University of Leicester, Leicester LE1 7RH, UK; kozaki@ono.co.jp (R.K.); m.vogler@kinderkrebsstiftung-frankfurt.de (M.V.); mjsd1@le.ac.uk (M.J.S.D.); 2 Ono Pharmaceutical Co. Ltd., Osaka 618-8585, Japan; 3 Institute for Experimental Cancer Research in Pediatrics, Goethe University Frankfurt, 60528 Frankfurt, Germany; 4 Ernest and Helen Scott Haematological Research Institute and Department of Cancer Studies, University of Leicester, Leicester LE1 7RH, UK; hw191@le.ac.uk (H.S.W.); sj179@le.ac.uk (S.J.); 5 MRC Toxicology Unit, University of Leicester, Leicester LE1 9HN, UK; dd5@le.ac.uk; 6 Institute for Human Genetics, Christian-Albrechts-University Kiel, D-24105 Kiel, Germany; reiner.siebert@uni-ulm.de; 7 Institute for Human Genetics, Ulm University, D-89081 Ulm, Germany; 8 University Hospital Schleswig Holstein, D-24105 Kiel, Germany

**Keywords:** DLBCL, BCR signaling, BTK, combination therapy

## Abstract

Bruton’s tyrosine kinase (BTK) is a key regulator of the B-cell receptor signaling pathway, and aberrant B-cell receptor (BCR) signaling has been implicated in the survival of malignant B-cells. However, responses of the diffuse large B-cell lymphoma (DLBCL) to inhibitors of BTK (BTKi) are infrequent, highlighting the need to identify mechanisms of resistance to BTKi as well as predictive biomarkers. We investigated the response to the selective BTKi, tirabrutinib, in a panel of 64 hematopoietic cell lines. Notably, only six cell lines were found to be sensitive. Although activated B-cell type DLBCL cells were most sensitive amongst all cell types studied, sensitivity to BTKi did not correlate with the presence of activating mutations in the BCR pathway. To improve efficacy of tirabrutinib, we investigated combination strategies with 43 drugs inhibiting 34 targets in six DLBCL cell lines. Based on the results, an activated B-cell-like (ABC)-DLBCL cell line, TMD8, was the most sensitive cell line to those combinations, as well as tirabrutinib monotherapy. Furthermore, tirabrutinib in combination with idelalisib, palbociclib, or trametinib was more effective in TMD8 with acquired resistance to tirabrutinib than in the parental cells. These targeted agents might be usefully combined with tirabrutinib in the treatment of ABC-DLBCL.

## 1. Introduction 

B-cell receptor (BCR) signaling has been implicated in the pathogenesis of some B-cell malignancies. The BCR is associated with *CD79A/B*, and it serves as a receptor to antigens to promote growth, proliferation, and survival of both normal and malignant B-cells. In normal B-cells, following antigen binding, the tyrosine kinases LYN and SYK initiate a signal transduction cascade involving adaptors, kinases, and second messengers, including the Bruton’s tyrosine kinase (BTK). This signaling pathway is subverted in diffuse large B-cell lymphoma (DLBCL) and other B-cell malignancies by mutations of key signaling molecules, including *CD79A/B* and *CARD11*.

BTK, a cytoplasmic tyrosine kinase of the tec protein tyrosine kinase (TEC) family, is an essential component of the BCR signaling pathway and plays an essential role in various biologic functions of different cell types. BTK mutations in humans leads to X-linked agammaglobulinemia (XLA) which is one of the most frequently inherited immunodeficiency diseases and is characterized by an almost complete arrest of B-cell differentiation at the pre-B cell stage. 

The introduction of the irreversible first-generation BTK inhibitor, ibrutinib, to clinical practice has changed outcomes for patients with chronic lymphocytic leukemia (CLL), mantle cell lymphoma (MCL), and Waldenström macroglobulinemia (WM) [1,2,3]. It binds strongly to cysteine 481 in the allosteric inhibitory segment of BTK kinase domain, resulting in the complete abrogation of BTK activity by inhibition of its autophosphorylation at tyrosine residue 223. However, the kinome of ibrutinib is broad [4,5] and may cause toxicities. Thus, several selective irreversible BTKi, including tirabrutinib (ONO/GS-4059), are now being assessed [6,7]; these appear to have comparable efficacy to ibrutinib but are associated with reduced toxicities [8] and will, therefore, have advantages in terms of combination therapies.

A subset of DLBCL display chronic active BCR signaling, which is not observed in resting B-cells that depend on tonic BCR signaling. This results in the constitutive activation of the NF-κB pathway. They are referred to as activated B-cell like lymphomas (ABC-like lymphomas). Individual knock-down of the different BCR components results in the death of the malignant B-cells. However, the clinical responses to BTKi are less satisfactory [9]. In a phase 1/2 study of ibrutinib in 80 patients with relapsed and refractory DLBCL (R/R DLBCL), responses were seen in 37% of ABC-DLBCL and 5% of germinal center B-cell like (GCB)-DLBCL [9]. The median response duration was 4.83 months (range: 1.02–9.26), although there were some long-term remitters. The acquisition of mutations within the BCR signaling pathway, present within the ABC-DLBCL subtype, had been thought to confer sensitivity to BTKi [9].

To improve outcomes with targeted therapies, in particular for R/R DLBCL which has a dismal prognosis, rational mechanism-based approaches to synergistic treatment combinations are key. A deeper understanding of the mechanisms conferring both primary and acquired resistance is required. We identified DLBCL lines sensitive to tirabrutinib and established a model of acquired resistance to tirabrutinib. The effectiveness of tirabrutinib combined with small molecules in six DLBCL cell lines and in acquired resistance to tirabrutinib was evaluated, and this evaluation indicated that the combination of tirabrutinib with idelalisib, palbociclib, or trametinib may be an effective treatment for ABC-DLBCL. 

## 2. Results

### 2.1. Anti-Proliferation and Apoptosis Activity

#### 2.1.1. The Anti-Proliferative Effect of Tirabrutinib in Hematopoietic Cell Lines

To assess the susceptibility of B-cell malignancies to tirabrutinib, a panel of 64 hematopoietic cell lines, including 10 GCB-DLBCL and 11 ABC-DLBCL lines, was screened (Table 1). Six showed a response to tirabrutinib when concentrations up to 10,000 nM were used; four of these were derived from ABC-type DLBCL (TMD8 with EC50 of 4.5 nM; OCI-LY10, U2932, and HBL1 each with an EC50 of approximately 3000 nM). The other responding cell lines were Pfeiffer, a GCB-DLBCL with an EC50 of around 3000 nM, and REC1, an MCL line with an EC50 of 33 nM [10]. The cell line most sensitive to tirabrutinib was the *CD79B* mutant cell line TMD8. However, other DLBCL cell lines containing the *CD79B* mutation (OCI-LY3, MD903) [11] were resistant, demonstrating that activating mutations in the BCR pathway do not necessarily predict a response. OCI-LY3 and MD903 cells also exhibit *CARD11* mutations [11], supporting previous observations that downstream *CARD11* mutations can block BTKi induced signaling [9].

EC50 was investigated after 72 h of exposure to tirabrutinib at 1 nM to 10,000 nM and an assessment of viability using CellTiterGlo^®^ (Promega Corp., Madison, WI, USA).

#### 2.1.2. Tirabrutinib Induced Cell Death in the TMD8 Cell Line

**Figure 1:**
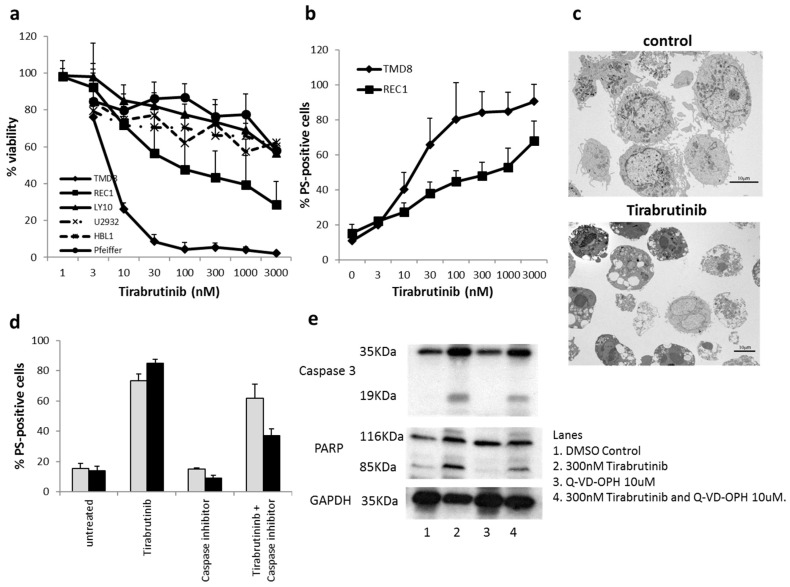
Effect of tirabrutinib on B-cell lines. (**a**) Six cell lines were exposed to different concentrations of tirabrutinib before an analysis of viability at 72 h using a CellTiterGlo^®^ Assay. Data shown here are the mean + SD of the six sensitive cell lines (*n* = 4–6). (**b**) TMD8 and REC1 cells were exposed to different concentrations of tirabrutinib for 72 h before analysis of apoptosis by AnnexinV-FITC staining by flow cytometry. Data shown are mean + SD (*n* = 6). (**c**) Electron microscopy of TMD8 cells left untreated (control) or exposed to 300 nM of tirabrutinib for 48 h. Cells display typical signs of apoptosis, including chromatin condensation, membrane blebbing, and mitochondrial changes. No other obvious morphologies were observed. (**d**) TMD8 cells were treated with 300 nM of tirabrutinib and/or the caspase inhibitor zVAD.fmk (25,000 nM, grey bars) or Q-VD-Oph (10,000 nM, black bars) for 72 h before analysis of apoptosis by AnnexinV-FITC staining by flow cytometry. Data shown are mean + SD (*n* = 3). (**e**) TMD8 cells were treated for 72 h before undergoing Western blot analysis.

Although viability was inhibited in all six sensitive cell lines (Figure 1a), TMD8 and REC1 underwent detectable apoptosis after exposure to tirabrutinib (Figure 1b). The cellular morphology assessed using electron microscopy confirmed classical apoptotic features (Figure 1c). The caspase inhibitors zVAD-fmk or Q-VD-OPhe reduced, but did not completely inhibit, cell death induced by tirabrutinib in TMD8 cells (Figure 1d), indicating that the type of cell death induced was partially caspase-independent. Furthermore, the onset of cell death induced by tirabrutinib was relatively slow, with a 72 h incubation being required to detect high levels of apoptosis. To investigate whether caspases are completely blocked under our experimental conditions we preformed Western blot analysis investigating the cleavage of caspase-3, as well as PARP as a caspase substrate (Figure 1e). While treatment with tirabrutinib clearly induced caspase-3 and PARP cleavage, PARP cleavage was only slightly reduced at 72 h of treatment with Q-VD-OPh, indicating residual caspase activity.

### 2.2. Mechanisms of Resistance to Tirabrutinib

**Figure 2:**
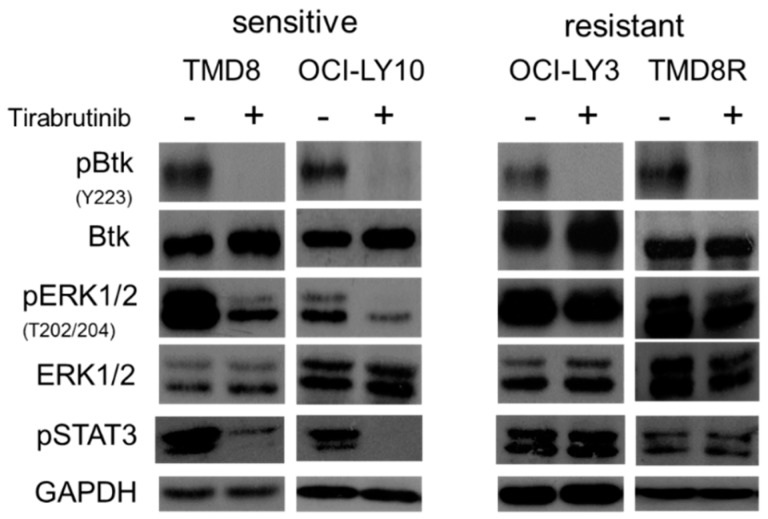
BTK inhibitor mechanism of resistance in ABC-DLBCL cell lines. Two sensitive (TMD8, OCI-LY10) and two resistant (OCI-LY3, TMD8R) cell lines were treated with 300 nM of tirabrutinib for 4 h. Cells were lysed and analyzed through Western blotting using anti-P-BTK, anti-P-ERK, anti-P-STAT3, and their non-phosphorylated forms. Data are representative of three individual experiments.

The phosphorylation of BTK was inhibited by tirabrutinib in sensitive and resistant cell lines, irrespective of whether they underwent cell death or not (Figure 2). However, the phosphorylation of extracellular signal-regulated protein kinases (ERK) was more strongly inhibited in the sensitive cell lines. In contrast, in both primary and secondarily tirabrutinib-resistant cell lines, OCI-LY3 and TMD8R, phosphorylation of ERK was maintained despite pY223BTK inhibition. TMD8R does not carry the BTK mutation C481S [12,13] but instead displays a mutation of PLCγ2 at R665W (Table 2) [12,13,14,15]. The efficacy (EC50) of tirabrutinib was reduced in the TMD8R cells (>30-fold). Additionally, phospho-STAT3 associated with cell survival and proliferation [16] was inhibited in the sensitive cells but maintained in the resistant cells, suggesting that the STAT3 signaling pathway might be a predictive marker of tirabrutinb sensitivity.

A Cys481S mutation of BTK prevents ibrutinib from covalently binding to the BTK mutants. It was found in patients with both CLL and MCL. As such, tirabrutinib is predicted to exhibit reduced binding and potency toward the Cys481S mutation compared to the wild type (WT), although it has not been reported to date in patients with tirabrutinib therapy. This secondary point mutation is a well-known mechanism of acquired drug resistance against kinase inhibitors. Specifically, point mutations of the gatekeeper often diminish the inhibitory activity of kinase inhibitors. However, a gatekeeper BTK mutant, BTK T474A, is sensitive to tirabrutinib (Table 3) but resistant to dasatinib (data not shown), which has a different mode of action from tirabrutinib and ibrutinib. On the other hand, the Cys481S mutation is sensitive to dasatinib. In addition to the Cys481S mutation, three distinct mutations in PLCγ2 were found in two CLL patients who became resistant to ibrutinib [12]. Two mutations in PLCγ2, R665W, and L845F could lead to a gain-of-function. Since PLCγ2 lies immediately downstream of BTK, these mutants could bypass the inactive BTK and allow autonomous B-cell receptor activity despite the inactive BTK. 

### 2.3. In Vivo Inhibition of Tumour Growth

**Figure 3:**
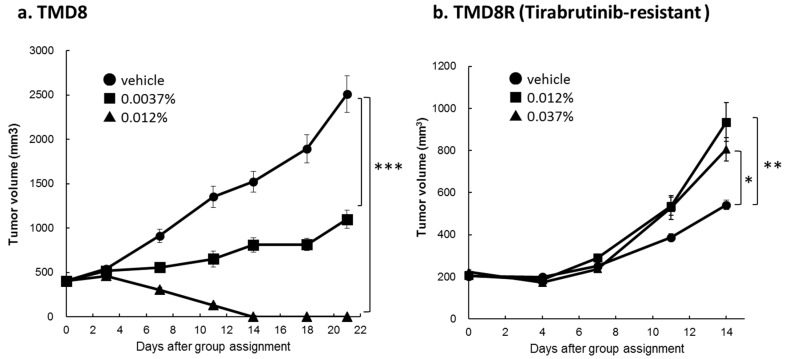
Anti-tumor activity of tirabrutinib in TMD8 and TMD8R xenograft models. SCID mice were injected with 1 × 10^7^ of (**a**) TMD8 or (**b**) TMD8R cells in Matrigel, subcutaneously. After randomization, mice were administered with a vehicle or tirabrutinib via mixed diet for three weeks (TMD8) or two weeks (TMD8R), respectively. The tumor volumes measured for nine mice for TMD8 and ten mice for TMD8R at each measurement time point are presented as the mean ± standard error. The Dunnett-test was used to compare data on tumor volume in the 0.0037%, 0.012%, and 0.037% group, versus the vehicle. A *p* value of less than 5% was considered statistically significant. * *p* < 0.05. ** *p* < 0.01. *** *p* < 0.001.

To confirm that tirabrutinib maintained activity in vivo, murine xenograft experiments were performed (Figure 3). A 0.012% treatment of tirabrutinib of parental TMD8 resulted in tumor remission in 10/10 animals (Figure 3a). In contrast, 0.012% and 0.037% treatment had no effect on tumor growth in tirabrutinib-resistant TMD8R cells (Figure 3b). When tirabrutinib was administered via mixed diet at an indicated relative content of 0.0037%, 0.012%, or 0.037%, the daily dosages for tirabrutinib were found to be comparable to the doses, 6, 20, or 60 mg/kg/day, respectively. No decreases in body weight were observed in any of the treatment groups [17].

### 2.4. Combination Activity

Next, we investigated whether combination treatments increased responses to tirabrutinib. Previous reports have indicated synergy between the BTKi ibrutinib and small molecules. However, some of these synergistic combinations may be due to off-target effects of ibrutinib and, hence, it is essential to perform detailed combination studies with the more specific BTKi tirabrutinib. Forty-three different drugs were combined with tirabrutinib in six DLBCL cell lines, including TMD8 and TMD8R (Table 4). Overall, there were few genuinely synergistic interactions with a combination index drug treatment (CI) of below 0.1, or very strong synergism, and these varied considerably between different cell lines. For example, in the HBL1 cell line there was potent synergistic interaction with the EZH2 inhibitor GSK-343 but not in any of the other cell lines. Three strongly synergistic effects in TMD8R were the PI3Kδ inhibitor idelalisib, the CDK4/6 inhibitor palbociclib, and the MEK inhibitor trametinib. This that these might be the mechanism of resistance in TMD8 cells (Table 3). Eight other targets, the pan-PIM inhibitor, the JAK2 inhibitor, the pan-PKC inhibitor, the BRD4 inhibitor, the mTORC1/2 inhibitor, and the immunomodulator lenalidomide also showed combination effects in TMD8R. However, each alone has a weak inhibitory effect on TMD8 viability.

**Figure 4:**
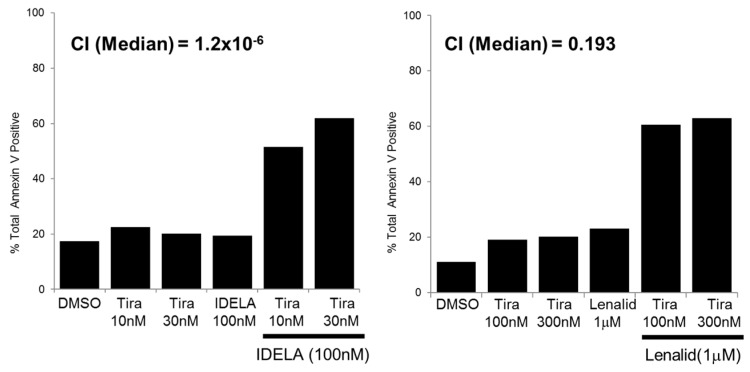
A combination of tirabrutinib and idelalisib or lenalidomide can overcome BTK-inhibitor resistance. TMD8R cells were treated with indicated doses of tirabrutinib combined with 100 nM of idelalisib or 1000 nM of lenalidomide for 72 h. Apoptosis was determined by AnnexinV-FITC staining by flow cytometry. The combination index was calculated using Calcusyn (Biosoft, Ferguson, MO, USA).

There might be different levels of apoptosis between the two distinctive pathways. In TMD8R, the combination of 100 nM of idelalisib and 30 nM of tirabrutinib induced 60% apoptosis as measured by annexinV staining, which were equivalent to the level of apoptosis observed in the lenalidomide combination (Figure 4). These data demonstrated that both pathways play a significant role, along with BTK signaling inhibition, in acquired tirabrutinib-resistant TMD8 cells, while the idelalisib combination shows strong synergy in non-resistant TMD8 cells. 

## 3. Discussion

Tirabrutinib is an irreversible second-generation BTK inhibitor with excellent efficacy and tolerability in R/R B-cell malignancies. Tirabrutinib forms a covalent bond with Cys481 of BTK and, like ibrutinib, irreversibly inhibits the kinase activity of BTK. Whilst clinical results with both drugs are particularly excellent in CLL, responses in DLBCL are usually only minor and short-lived, indicative of both primary and acquired resistance. Rational, mechanism-based combinations are required to be taken into the clinic to improve the depth and duration of responses. Consistent with previous studies with ibrutinib, responses to tirabrutinib were enriched in ABC-DLBCL cell lines. However, not all ABC-DLBCL cell lines were sensitive, despite the presence of *CD79B* mutations, suggesting that additional BCR independent mechanisms exist to drive tumor genesis, including chronic active BCR signaling occurring due to non-genetic mechanisms, as previously proposed by Wilson et al. [9] and seen in the context of CLL, MCL, and WM.

In the present study, tirabrutinib-resistant TMD8 which had PLCγ2 R665W was examined by combination therapy. It has been reported that tirabrutinib in combination with idelalisib, entospletinib, and the BCL2 inhibitor ABT-199 synergistically increased apoptosis in primary CLL cells, a subset of DLBCL and MCL cell lines [10,18,19]. Interestingly, the BTK Cys481S mutation activated cell-cycle reprogramming besides AKT activation [20], while the CDK4/6 inhibitor palbociclib, in combination with tirabrutinib, showed strong synergy in our resistant model, TMD8R, which does not have BTK Cys481S. Additionally, previous studies on the synthetic lethality of ibrutinib in ABC-DLBCL have been explored [21,22]. In this study, we found that the MEK1/2 inhibitor trametinib showed strong synergy with tirabrutinib in the acquired resistant TMD8. Western blotting analysis clearly interpreted this effective combination where ERK was highly up-regulated in TMD8R. These suggested that the combination therapy may overcome some mechanisms of resistance in the BTK signaling pathway and supported the clinical investigation of these combinations in patients with CLL and B-cell lymphoma. 

From a clinical perspective, the challenge lies in identifying biomarkers that would predict responses within each disease subtype and developing in vivo models that would enable early response and relapse to be predicted. Responses, when seen, are often brief and, therefore, rational combinations are required to take forwards into the clinic, to improve the depth and duration of response. We have shown that, of the compounds tested in vitro, idelalisib, palbociclib, and trametinib may prove the most promising and warrant further investigation. The synergistic mechanisms of idelalisib or entospletinib, in combination with tirabrutinib, are currently being explored in the phase 1b combination study GS-US-401-1757 (NCT02457598).

## 4. Materials and Methods

### 4.1. Cell Death Assessment

The cell lines used in this study have been previously described and were obtained either from the originators or from Deutsche Sammlung von Mikroorganismen und Zellkulturen GmbH (DSMZ). The identity of cell lines were confirmed by both metaphase cytogenetics and short tandem repeat assessment. Cell lines were grown in RPMI 1640 supplemented with 10% fetal calf serum. Sensitivity to tirabrutinib in cell lines as well as combination treatments was performed using a CellTiterGlo^®^ viability assay or AnnexinV-FITC staining. The calculation of EC50 was done using GraphPadPrism. Tirabrutinib resistant TMD8 (TMD8R) was generated by continuous exposure over nine months at concentrations of tirabrutinib ranging from 3 nM to 1000 nM until stable resistance to tirabrutinib was established. Specifically, the concentration of tirabrutinib was gradually increased from 3, 6, 12, 25, 40, 60, 80, 100, 200, 400, 800, and 1000 nM. Cell passage was performed twice a week. When the growth was fine, cells were re-suspended with fresh media containing the same concentration of tirabrutinib (final cell density was 100,000 cells/mL). Sensitivity of cells to tirabrutinib was tested by CellTiterGlo^®^ at every step of passage. The mutational status of TMD8R was determined by Sanger sequencing. The combination index was calculated using CalcuSyn based on the multiple drug-effect equation of Chou-Talalay.

### 4.2. Immunoblot Assays

Treated or untreated cells were lysed in Radio-Immunoprecipitation assay (RIPA) buffer containing a protease inhibitor cocktail. Western blotting was performed using antibodies from Cell Signaling for BTK, ERK, and STAT3 and Novus Biologicals for phospho-BTK.

### 4.3. Mouse Xenograft Model

To assess in vivo efficacy of tirabrutinib, severe combined immunodeficiency (SCID) mice were injected with 1 × 10^7^ cells in Matrigel, subcutaneously. Randomization and treatment were initiated when the mean tumor volumes reached 400 mm^3^ for TMD8 and 200 mm^3^ for tirabrutinib resistant cells. Groups of mice were then dosed via diet containing tirabrutinib at concentrations of 0.0037%, 0.012% and 0.037%. The daily dosage was found to be comparable to the doses, 6, 20, and 60 mg/kg/day, respectively. Tumor growth was assessed using a caliper.

## 5. Conclusions

We investigated the response to tirabrutinib in a panel of 64 hematopoietic cell lines. Although ABC-DLBCL cells were most sensitive amongst all cell types studied, the sensitivity did not correlate with BCR pathway mutations. We investigated combination strategies with 43 drugs in DLBCL. Tirabrutinib combined with idelalisib, palbociclib, or trametinib was more effective in ABC-DLBCL with acquired resistance to tirabrutinib than in the parental cells. Taken together, our data indicates that these targeted agents might be usefully combined with tirabrutinib in the treatment of ABC-DLBCL.

## Figures and Tables

**Table 1:** EC50 for tirabrutinib in 64 hematopoietic cell lines.

Cell Line	Type	Mutations	EC50 for Tira (nM)
TMD8	ABC-DLBCL	CD79A/B, MYD88, PIM1, IRF4, MYC, PRDM1	4.5
OCI-Ly10	ABC-DLBCL	CDK11B, PTEN, TP53, TP63, CARD11	~3000
OCI-Ly3	ABC-DLBCL	CDK11B, CD79B, MYD88, BCL6, TP63, IRF4, PIM1, CARD11	>3000
MD901	DLBCL	n.d.	>3000
MD903	DLBCL	CD79B, BCL6, CARD11, PIM1	>3000
U2932	ABC-DLBCL	TP53	~3000
HBL1	ABC-DLBCL	CD79, CARD11, MYD88	~3000
NU-DUL-1	ABC-DLBCL	TP53, TP63, KRAS, PLCG2, MYC, ATR	>3000
RC-K8	ABC-DLBCL	RELN, CARD11	>3000
RIVA/RI-1	ABC-DLBCL	NOTCH2, CDK18, TP53, TP63, FOXO4	>3000
RS-27	ABC-DLBCL		>3000
DB	GCB-DLBCL		>3000
Pfeiffer	GCB-DLBCL		~3000
OCI-Ly19	GCB-DLBCL		>3000
SU-DHL4	GCB-DLBCL		>3000
SU-DHL6	GCB-DLBCL		>3000
SU-DHL10	GCB-DLBCL		>3000
DOHH2	GCB-DLBCL, tr F		>3000
Boer88	GCB-DLBCL, tr F		>3000
SC1	GCB-DLBCL, tr F		>3000
KARPAS422	GCB-DLBCL, tr F		>3000
Karpas 1106	PMBCL		>3000
MedB-1	PMBCL		>3000
KIS-1	DLBCL		>3000
REC1	MCL		33
GRANTA-519	tr MCL		>3000
JEKO-1	MCL		>3000
MINO	MCL		>3000
JVM2	MCL		>3000
MUTZ5	BCP-ALL		>3000
TANOUE	BCP-ALL		>3000
KASUMI-2	BCP-ALL		>3000
NALM-6	BCP-ALL		>3000
RCH-ACV	BCP-ALL		>3000
REH	BCP-ALL		>3000
SEM	BCP-ALL		>3000
KOPN-8	BCP-ALL		>3000
380	BCP-ALL		>3000
CEMO1	BCP-ALL		>3000
NALM-27	BCP-ALL		>3000
PER-377	BCP-ALL		>3000
LK63	BCP-ALL		>3000
LILA-1	BCP-ALL		>3000
MHH-CALL-4	BCP-ALL		>3000
697	BCP-ALL		>3000
MEC1	CLL		>3000
MEC2	CLL		>3000
EHEB	CLL		>3000
HL60	AML		>3000
MV 411	AML		>3000
BV173	CML		>3000
K562	CML		>3000
MEG01	CML		>3000
NALM-1	CML		>3000
LP1	MM		>3000
RPMI 8226	MM		>3000
U266	MM		>3000
SSK41	MZL		>3000
Daudi	BL		>3000
Raji	BL		>3000
Ramos	BL		>3000
NAMALWA	BL		>3000
Jiyoye	BL		>3000
GUMBUS	BL		>3000

PMBCL, Primary mediastinal large B-cell lymphoma; BCP-ALL, B-cell precursor acute lymphoblastic leukemia; AML, Acute myeloid leukemia; CML, Chronic myeloid leukemia; MM, Multiple myeloma; MZL, Marginal zone lymphoma; BL, Burkitt lymphoma.

**Table 2:** Genetic background of BTK inhibitor resistance in TMD8R cells.

Proteins	Mutations	TMD8R
BTK	C481S [12,13]	No
BTK	C481R [14]	No
BTK	T316A [15]	No
PLC 2	L845F [12]	No
PLC 2	R665W [12]	Yes
PLC 2	S707Y [12]	No
PLC 2	Y495H [14]	No

Genomic DNA was extracted from TMD8R. BTK and PLCγ2 genes were sequenced in order to confirm whether the cell line has the acquired mutations observed with ibrutinib in CLL.

**Table 3:** Interaction kinetics with BTK WT and BTK T474A.

Test Compound	BTK WT Kd (nM)	BTK T474A Kd (nM)
Tirabrutinib	12.5	1.87

Kd assessed after 2 h tirabrutinib treatment with target protein.

**Table 4:** Combination index for tirabrutinib.

Drug	Target	TMD8	LY10	U2932	HBL-1	LY3	Pfeiffer	TMD8R
AZD-1208	pan-PIM	0.67	0.32	0.22	0.12	0.55	0.48	0.39
ABT-737	Bcl-xL, Bcl-2, Bcl-w	0.11	0.71	0.28	>1	0.41	n.d.	n.d.
Idelalisib	PI3K	0.17	0.16	>1	>1	0.21	0.74	0.02
GSK-343	EZH2	>1	0.83	0.24	0.01	>1	0.46	>1
TG101348	JAK2	0.64	0.83	0.26	0.59	0.72	1	0.16
Sotrastaurin	pan-PKC	0.49	0.76	>1	0.44	>1	0.73	0.38
MLN-4924	NAE	0.32	1	0.11	>1	>1	>1	0.70
NVP-LDE-225	SMO	0.72	>1	0.58	>1	0.28	>1	>1
Selumetenib	MEK1,2	0.73	0.72	>1	>1	0.23	>1	0.15
CPI203	BRD4	0.21	0.64	>1	>1	>1	>1	0.18
MALT-MI-2	MALT-1	>1	0.69	>1	0.44	0.97	0.92	0.83
PPT	ER agonist	>1	>1	>1	0.12	>1	>1	n.d.
BX912	PDK1	0.76	n.d.	>1	0.62	>1	0.92	>1
GDC-0349	mTORC1/2	0.49	0.67	>1	>1	>1	>1	0.20
Chloroquine	Lysosome	0.89	0.94	0.89	0.53	>1	>1	0.90
AT9283	AuroraA/B, JAK2/3	>1	1	>1	0.54	0.76	>1	n.d.
GSK-461364	PLK1	>1	>1	0.33	>1	>1	>1	>1
4HPR	Retinoid derivative	>1	>1	>1	0.5	>1	>1	>1
Dinaciclib	CDK1/2/5/9	>1	0.94	0.6	>1	>1	>1	>1
Panobinostat	pan HDAC	>1	>1	>1	0.56	>1	>1	n.d.
Volasertib	PLK1,2,3	1	>1	0.59	>1	>1	>1	>1
Olaparib	PARP1	0.61	>1	>1	>1	>1	>1	>1
BMS-911543	JAK2	>1	>1	>1	>1	>1	0.67	0.46
SKP2-C25	SKP2	>1	0.71	>1	>1	>1	>1	n.d.
NVP-AUY-922	Hsp90	>1	0.72	>1	>1	>1	>1	>1
Carfilzomib	Proteasome	>1	0.73	>1	>1	>1	>1	0.62
2-DG	Glycolytic pathway	0.93	n.d.	>1	1	>1	0.88	>1
CX5461	rRNA synthesis	0.84	n.d.	>1	>1	>1	>1	0.96
Palbociclib	CDK4/6	0.82	0.99	>1	>1	>1	>1	0.07
Desipramine	Lysosome	0.85	0.99	>1	>1	>1	>1	n.d.
CID755673	PKD	>1	>1	0.86	>1	>1	>1	>1
R428	AXL	0.95	0.93	>1	>1	>1	>1	n.d.
Lenalidomide	Immunomodulatory	0.9	>1	>1	>1	>1	>1	0.42
RG-7388	MDM2-p53	0.92	n.d.	>1	>1	>1	>1	0.87
FTY720	S1P agonist	1	0.93	>1	>1	>1	>1	n.d.
Ibrutinib	BTK, HER2	0.96	>1	>1	>1	>1	>1	>1
Imatinib	BCR-ABL	0.94	>1	>1	>1	>1	>1	0.77
Trametinib	MEK1,2	>1	>1	>1	>1	>1	>1	0.09
PLX4720	BRAF	>1	>1	>1	>1	>1	>1	n.d.
Enzastaurin	PKC	>1	>1	>1	>1	>1	>1	>1
TRAM-34	KCa3.1	>1	>1	>1	>1	>1	>1	n.d.
PAP-1	Kv1.3	>1	>1	>1	>1	>1	>1	>1
DPN	ER agonist	>1	>1	>1	>1	>1	>1	n.d.

Seven DLBCL cell lines (one sensitive, four partially responsive, and two resistant) were exposed to different concentrations of tirabrutinib combined with 34 different targeted inhibitors for 72 h before assessment of viability using CellTiterGlo^®^. The combination index was calculated using Calcusyn (Biosoft, Ferguson, MO, USA). Data shown are the median combination indices of all concentrations tested.
